# When size matters: Managing a giant pulmonary hydatid cyst in a pediatric patient: Case report

**DOI:** 10.1097/MD.0000000000044212

**Published:** 2025-09-12

**Authors:** Haya Abu Mayyaleh, Bayyena Abu-Radwan, Islam Jadallah, Mohammed Saleh, Ahmad Fasfoos, Yousef Abu Asbeh

**Affiliations:** a Faculty of Medicine, Hebron University, Hebron, Palestine; b Department of thoracic surgery, Al-Ahli Hospital, Hebron, Palestine.

**Keywords:** case report, hydatid cyst, hydatid disease, pediatric surgery, video-assisted thoracoscopic surgery

## Abstract

**Rationale:**

Giant pulmonary hydatid cysts in children are rare, with nonspecific symptoms often delaying diagnosis. Preservation of lung parenchyma during surgery is essential. This is the first reported case of such a cyst in a Palestinian pediatric patient.

**Patient concerns:**

A 5-year-old girl presented with progressive left flank pain, dyspnea, and fever; initially misdiagnosed as gastroenteritis.

**Diagnoses:**

Chest X-ray and computed tomography revealed a hydatid cyst occupying >90% of the left lower lung lobe with pleural effusion.

**Interventions:**

Urgent lateral thoracotomy combined with video-assisted thoracoscopic surgery was performed for cyst evacuation and sterilization, preserving lung parenchyma.

**Outcomes:**

Successful removal with uneventful recovery; discharged in stable condition after 6 days.

**Lessons:**

Early imaging and diagnosis are critical. Hybrid surgical approaches can remove large cysts while preserving lung tissue. Hydatid disease should be considered in children with unexplained respiratory or gastrointestinal symptoms in endemic regions.

## 1. Introduction

This case report has been prepared in accordance with the 2013 CARE statement article.^[1]^

Hydatid cyst (HC), a zoonotic disease caused by *Echinococcus granulosus*, is prevalent in regions with extensive livestock farming, including the Mediterranean, South America, Central Asia, and parts of Africa.^[2]^ The overall prevalence of hydatid cysts in Palestine is reported to be 9%.^[3]^ Humans acquire the disease through ingestion of eggs from infected animals.^[4]^ HC most commonly affects the liver in adults (60%–80% of cases) and the lungs (20%–30%)—although it can also involve the brain, kidneys, and musculoskeletal system.^[5–7]^ In pediatric cases, the lungs are frequently affected, with the right lower lobe being the most commonly involved site.

Clinically, small cysts are typically asymptomatic; however, larger cysts may present with respiratory symptoms such as cough, dyspnea, and chest pain.^[4]^ If left untreated, complications such as cyst rupture and secondary bacterial infections may arise.^[8,9]^ Macroscopically, hydatid cysts are typically spherical, unilocular, and filled with clear fluid. Microscopically, they consist of an outer lamellar layer and an inner germinal layer, often containing daughter cysts.

Diagnosis is primarily based on imaging studies—particularly computed tomography (CT) scans—and serologic tests, such as enzyme-linked immunosorbent assay, although false negatives can occur.^[10,11]^ Other differential diagnoses may include pulmonary abscess, bronchogenic cyst, or primary lung tumors.^[12]^ Treatment options usually range from conservative or radical surgical approaches to minimally invasive procedures, such as puncture, aspiration, instillation, and respiration.^[13]^

We present a case of a 5-year-old female patient who presented with left-sided flank pain associated with a cough, dyspnea, and fever. Radiologic imaging revealed a large hydatid cyst occupying the majority of the left lower lung lobe. This finding represents the first reported case in Palestine of a hydatid cyst of a significant magnitude in a pediatric patient, presented with nonspecific symptoms which can often be attributed to a broad spectrum of conditions. Notably, the patient did not present with any identifiable risk factors commonly associated with hydatid disease. This case highlights the critical importance of early diagnosis and appropriate management for pediatric patients with hydatid cysts.

## 2. Case presentation

A 5-year-old Palestinian girl presented to an outpatient clinic with progressive left-sided flank pain persisting for 2 months. The pain was stabbing in nature and worsened over time, accompanied by intermittent fever (up to 38°C). Initially misdiagnosed as gastroenteritis, she was treated with intravenous normal saline and analgesia; however, her symptoms persisted despite treatment. In the week prior to admission to our private medical facility, the patient became increasingly hypoactive and developed anorexia. Additional symptoms included dyspnea on exertion, a productive cough with yellowish sputum, daily fever, and chills. She had no significant family history of hydatid disease or other chronic illnesses and lived in an urban setting with no direct exposure to livestock. Prior to this episode, there were no significant hospitalizations recorded. On physical examination, decreased air entry was noted on the left side of the chest, while vital signs and the remainder of her examination were unremarkable. Laboratory tests indicated anemia (hemoglobin level of 9.3 g/dL), leukocytosis (WBC 24.1 × 10³/µL, 88.6% neutrophils), thrombocytosis (platelets of 500 × 10³/µL), and an elevated C-reactive protein (140.1 mg/L). Liver function tests were within normal limits, aside from a total bilirubin of 1.5 mg/dL. Electrolyte levels were normal, with the exception of mild hyperchloremia (118 mmol/L).

**Figure 1.**
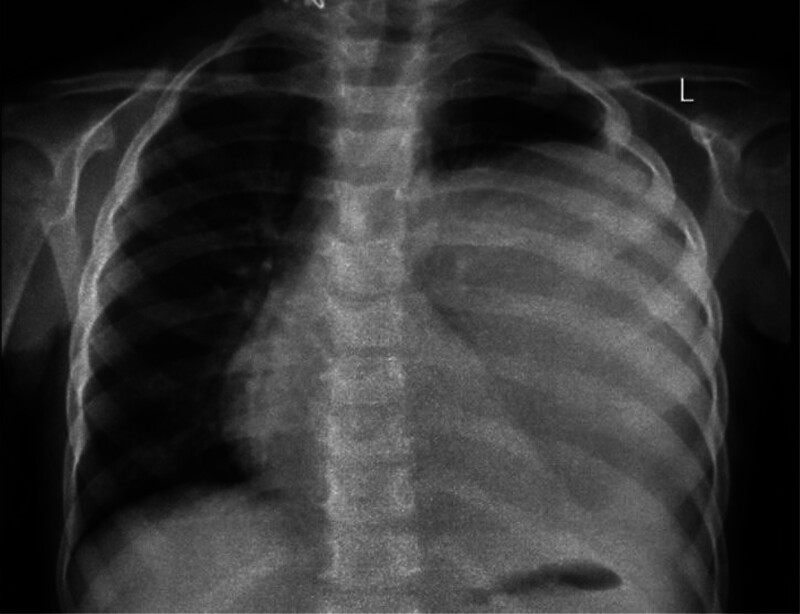
Chest X-ray (PA view) demonstrates a large lesion occupying most of the left hemithorax, with a noticeable mediastinal shift to the contralateral side. There is also depression of the ipsilateral diaphragm, suggesting significant space-occupying pathology in the left lung.

**Figure 2.**
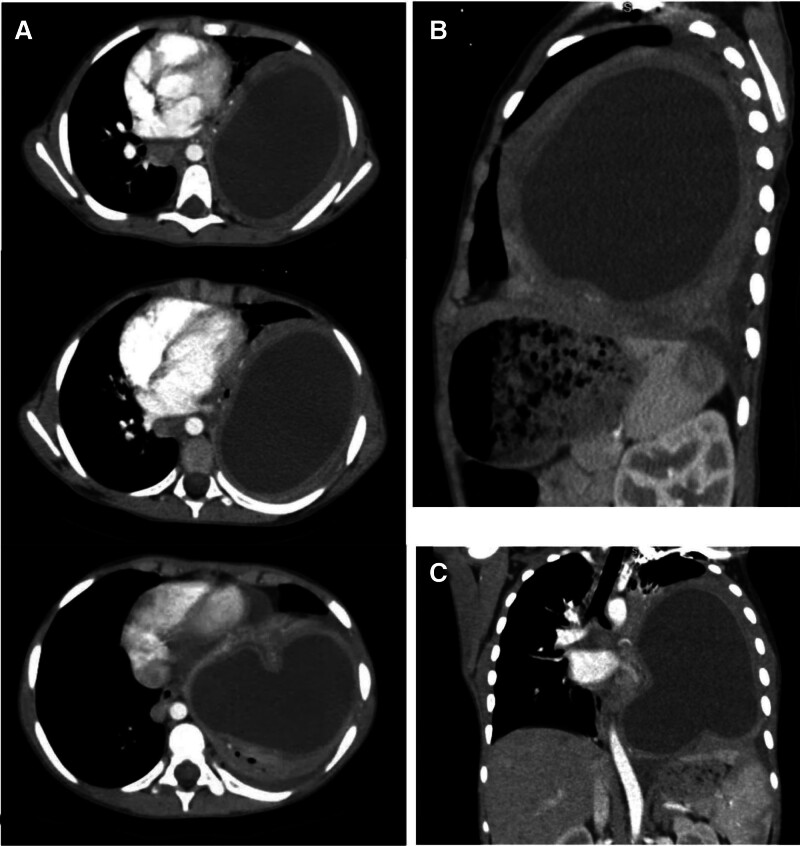
Chest CT scan with IV contrast (mediastinal window) shows a huge cystic lesion occupying most of the left hemithorax, with a marked mediastinal shift to the contralateral side. The cyst demonstrates fluid density, consistent with a large hydatid cyst or other cystic lesion. Significant compression of the surrounding structures, including the ipsilateral lung and diaphragm, is noted. (A) axial view, (B) sagital view, (C) coronal view.

The patient was treated with intravenous Ceftriaxone 1 g every 12 hours, Morphine 0.1 mg/kg/dose, Metronidazole 500 mg every 8 hours, and oxygen via nasal canula (2 L/min). Imaging studies revealed a large, well-defined cystic lesion occupying the majority of the left lower lung lobe. A chest X-ray demonstrated a dense, homogeneous opacity (Fig. 1). A contrast-enhanced CT scan confirmed a thick-walled cystic lesion (9 × 8.5 × 11 cm) with surrounding lung consolidation, mild pleural effusion, and a 2.7 × 1.1 cm subcarinal soft tissue density (Fig. 2). These findings were suggestive of either a lung abscess or a hydatid cyst. Serological testing was not performed due to financial constraints. The delay in diagnosis occurred as a result of the patient’s general and nonspecific symptomology, compounded by an initial misdiagnosis.

Given the size of the cyst and associated complications, urgent surgical resection was performed by a Consultant Thoracic Surgeon with extensive expertise in thoracic surgery and minimally invasive procedures. The patient was placed in the lateral decubitus position under general anesthesia with single-lumen right-sided intubation. She underwent an urgent lateral thoracotomy using a muscle-sparing approach via the serratus anterior and video-assisted thoracoscopic surgery (VATS). A large hydatid cyst occupying nearly the entire left lower lung lobe was identified, along with significant adhesions and compression of residual lung tissue. Initially, 350 cc of fluid was aspirated from the superior portion of the cyst, followed by the instillation of 160 cc of 23% hypertonic saline for sterilization.

**Figure 3.**
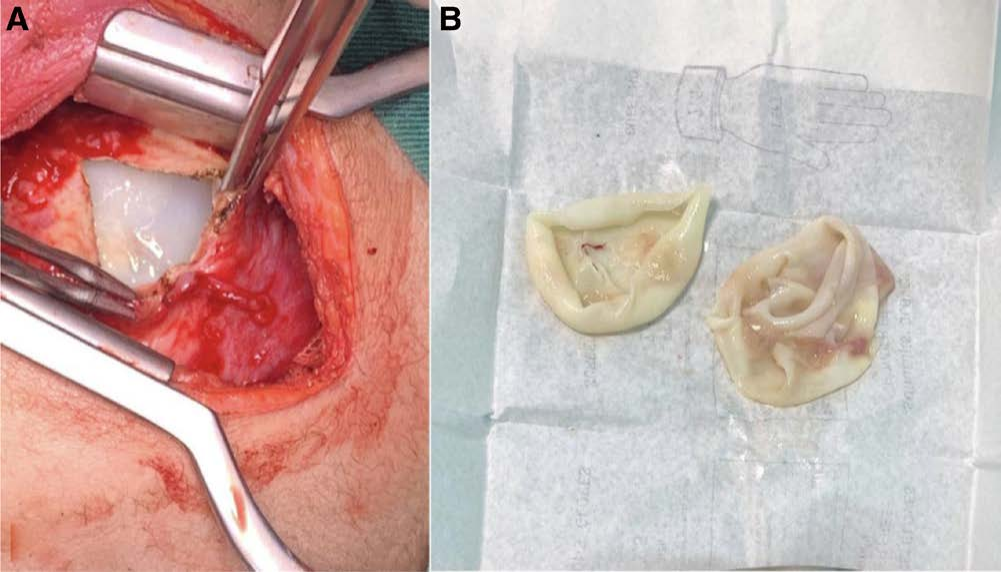
(A) Intraoperative images of the hydatid cyst demonstrate the minithoracotomy incision used for access. The germinal layer of the cyst is clearly visible in the surgical field. (B) The resected germinal layer.

Complete isolation of the cyst and release of lung adhesions were successfully achieved. The germinative membrane was excised from the cyst through cystotomy, after widening the needle insertion site (Fig. 3). The residual cavity was then carefully cleaned and reexamined for the presence of possible daughter vesicles. Despite involvement of more than 90% of the left lower lobe, lobectomy was avoided. Four bronchial openings were identified and closed using Vicryl sutures. The cavity was marsupialized and closed with multiple sutures. The procedure concluded with thorough irrigation, insertion of a chest tube, and layered closure of the thoracic wall. The patient was extubated on the operating table.

**Figure 4.**
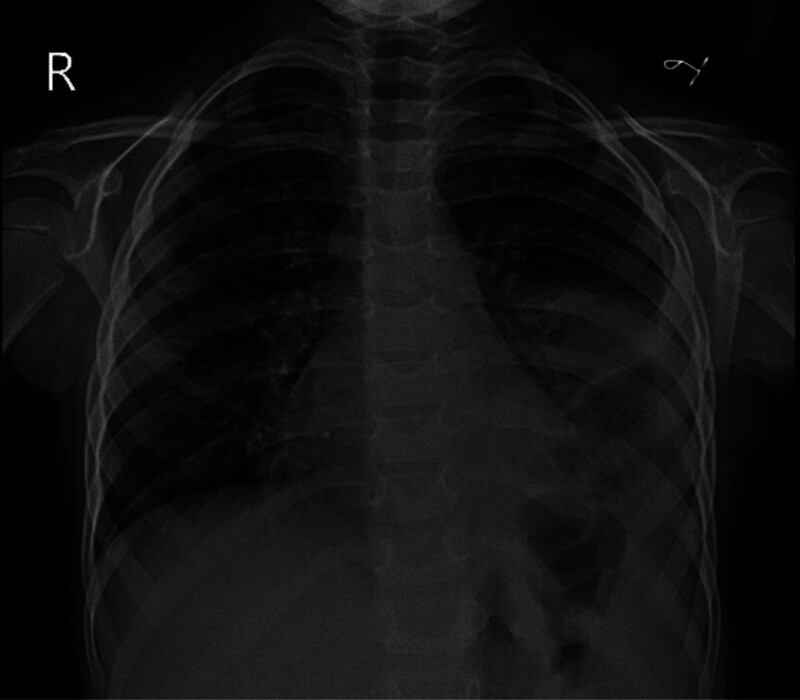
Postoperative chest X-ray (PA view) shows resolution of the previously visualized large cystic lesion. The chest tube has been removed, and a mild residual pleural effusion is noted. No significant abnormalities are observed, and the lung fields appear clear, indicating favorable postsurgical recovery.

The patient was transferred to the pediatric intensive care unit for close postoperative monitoring. Her recovery was uneventful, with rapid clinical improvement. By the second postoperative day, she was transferred to the surgical department. The chest tube was removed on postoperative day 5, and she was discharged in stable condition on day 6, which is within the expected range for such procedures in pediatric patients, with a prescription for Albendazole 130 mg for 30 days. A chest X-ray at discharge confirmed satisfactory lung expansion (Fig. 4).

At her 2-week follow-up visit, the patient remained clinically stable with a well-healed chest wound. Over 6 months of follow-up, she continued to do well in good health, with no recurrence. The parents expressed gratitude for the medical team’s efforts in ensuring a smooth recovery and providing ongoing support. In light of the uneventful postoperative course and full recovery, the outcome was categorized as Clavien-Dindo Grade 0, indicating an expected recovery without complications or need for additional intervention. The chronological sequence of the patient’s symptoms, interventions and outcomes is summarized in Table 1.

**Table 1:** Clinical timeline of the patient’s presentation, management and follow-up.

Timeframe	Event
2 mo before admission	Onset of progressive left-sided flank pain (stabbing in nature), gradually worsening over time.
Early symptoms	Developed fever (38°C), initially misdiagnosed as gastroenteritis and treated with IV fluids and analgesia. Symptoms persisted.
1 wk before admission	Symptoms worsened: hypoactivity, anorexia, dyspnea on exertion, productive cough (yellow sputum), daily fever, and chills.
Day of hospital admission	Diagnosis of a large hydatid cyst occupying most of the left lower lung lobe was confirmed.
Day 1—Surgery	Urgent lateral thoracotomy and video-assisted thoracoscopic surgery performed.
Post-op day 1	Extubated on the table after surgery. Transferred to PICU for monitoring.
Post-op day 2	Transferred to the surgical department; remained in stable condition.
Post-op day 5	Chest tube removed.
Post-op day 6 (discharge)	Discharged home in stable condition.
6-mo follow-up	No recurrence. Parents reported full recovery and expressed gratitude.

PICU = pediatric intensive care unit, IV = intravenous.

## 3. Patient perspective

The patient’s parents expressed deep gratitude for the medical team’s dedication and expertise in managing their child’s condition. They specifically highlighted the compassionate care provided throughout hospitalization and follow-up period, emphasizing the importance of clear communication and emotional support. Over 6 months of follow-up, they reported that their child remained in good health, with no recurrence of symptoms, reinforcing their confidence in the treatment approach.

## 4. Discussion

Echinococcosis, or hydatid disease, is a parasitic infection caused by the larval stage of *Echinococcus granulosus*. It remains a significant public health concern, particularly in developing regions such as Asia, the Mediterranean, South America, and Africa.^[2]^ According to the World Health Organization, prevalence rates in endemic areas such as Argentina, Peru, East Africa, Central Asia, and China range between 5% and 10%. In Palestine, an observational case series of surgically confirmed cases reported an incidence rate of 2.1 per 100,000 in the West Bank and 0.13 per 100,000 in the Gaza Strip, with an overall average of 1.1 per 100,000.^[3]^

The life cycle of Echinococcus involves a predator–prey relationship, where definitive hosts (e.g., dogs) harbor the adult worm and excrete eggs in their feces. Intermediate hosts, such as sheep ingest these eggs, leading to larval migration into various organs. Humans become accidental hosts through ingestion of contaminated food, water, or direct contact with infected animals.^[4]^

The liver is the most commonly affected organ (75% of cases),^[14]^ as it serves as the first filter for parasitic embryos. When larvae bypass the liver, they can reach the lungs (the second most common site) or, less commonly, other systemic organs.^[5]^ Multiple organ involvement occurs in 10.3% of cases, with the liver–lung combination being the most frequent (7.7%).^[6]^ Hydatid cysts account for roughly 7% of pediatric pulmonary infections globally, while soft tissue involvement is extremely rare, (only < 1%) in pediatric patients.^[7]^ This rarity may be attributed to the muscular contractions and the production of lactic acid, which may impede cyst development and growth in muscle tissue.^[15]^

In Palestine, variations in living conditions—urban, rural, and refugee settings—have a noticeable impact on healthcare access and disease patterns. Demographically, HC cases are most common among villagers (78%), followed by city-dwellers (20%), refugee camp residents (2%), and Bedouins living in encampments (0.3%). Cultural practices, such as keeping dogs and livestock in household environments, increase the risk of Echinococcus granulosus transmission through intermediate hosts.^[3]^

Clinically, hydatid disease often presents in a nonspecific manner. The disease may remain asymptomatic for many years, with symptoms appearing only once cysts enlarge, compressing affected and nearby organs. Clinical presentation varies depending on cyst location, complicating diagnosis.^[4]^ Several reports emphasize delayed or missed diagnoses, due to atypical cyst locations or secondary infections that obscure clinical findings.^[8]^

Imaging is essential for the diagnosis of hydatid cysts, particularly due to their distinctive radiologic features. Ultrasonography is a valuable, noninvasive modality for detecting hydatid disease. The CT appearance of hydatid cysts can vary and may resemble solid tumors due to inflammatory changes. MRI plays an important role in delineating the extent of involvement of skeletal muscle, excluding differential diagnoses, and guiding surgical planning.^[10]^ Chest X-ray is the initial modality of imaging used in pulmonary hydatid cysts, while CT and MRI provide superior anatomical detail and diagnostic accuracy.^[11]^

Treatment options for soft tissue hydatid disease include surgery, chemotherapy, and the puncture, aspiration, instillation, and respiration technique.^[13]^ Surgical cystectomy is the primary modality of treatment, which should be radically performed to avoid any spillage of the cyst contents.^[16]^ Surgery is the treatment of choice for pulmonary hydatid cysts, and it can safely be performed with very minimal morbidity and almost no mortality, irrespective of the size of the cysts.^[17]^ In our case, a hybrid technique of lateral thoracotomy combined with VATS was chosen over a purely minimally invasive approach due to the lesion’s size and complexity, requiring optimal exposure and manual palpation for complete resection. This hybrid approach provided the visualization benefits of VATS alongside the control and flexibility of thoracotomy, ensuring safe and effective management of the case.

The differential diagnosis of an infected hydatid cyst includes abscesses and neoplasms as its high density may mimic hydatid cysts on imaging.^[12]^ Differential diagnoses for uncomplicated hydatid cysts on chest X-rays include inflammatory masses, fluid-filled cysts, benign tumors, carcinoma, and metastases. A radiographic crescent sign, commonly associated with hydatid cysts, may also appear in conditions such as aspergilloma, cavitating malignancies, blood clots, and Rasmussen aneurysm.^[11]^

This case highlights the diagnostic challenges of hydatid disease in pediatric patients, particularly in endemic regions such as Palestine. The initial misdiagnosis of gastroenteritis—based on nonspecific symptoms such as abdominal discomfort and fever—led to delayed recognition of the hydatid cyst. This delay, compounded by inappropriate treatment, allowed the cyst to grow to a significant size, occupying nearly the entire left lower lobe of the lung. Such delays not only increase the risk of complications, including rupture or secondary infection, but also make surgical management more challenging due to the extensive involvement of lung tissue.

Our review identified several documented cases of pulmonary hydatid cysts in young children that were initially misdiagnosed. One such case, reported in 2019, involved a 9-year-old diagnosed and treated for pulmonary hydatid cysts after an initial presumptive diagnosis of bacterial lung abscess.^[8]^ Another case from 2024 described a superimposed infected pulmonary hydatid cyst in a 7-year-old, initially misdiagnosed as complicated pneumonia.^[18]^ A case from 2018 involved a 10-year-old diagnosed with pulmonary hydatid disease after presenting with symptoms of rapidly progressing necrotizing pneumonia.^[19]^ Like these cases, our patient initially received a misdiagnosis; however, the cyst’s extreme size and successful lung-preserving surgical approach distinguish this case and add to the existing literature.

This case provides valuable insights into the diagnosis and management of giant pulmonary hydatid cysts in pediatric patients. However, certain limitations should be acknowledged. While the patient demonstrated a stable recovery over a 6-month period, extended follow-up would be beneficial to evaluate the potential for recurrence or delayed complications. Another limitation is the absence of serological testing, which could have further confirmed and supported the diagnosis but was not performed due to financial constraints. This highlights the challenges encountered in resource-limited settings, where access to comprehensive diagnostic tools may be restricted, potentially leading to diagnostic delays or reliance on imaging for diagnosis.

## 5. Conclusion

This case highlights the critical importance of early recognition of hydatid disease in pediatric patients, particularly in endemic regions such as Palestine. The atypical presentation, diagnostic challenges, and the successful implementation of a hybrid surgical approach make this case especially unique. Notably, it represents the first documented case in Palestine of such a large hydatid cyst, at this scale, in a pediatric patient of this age group. This underscores the need for heightened clinical suspicion, timely imaging, and tailored surgical strategies to achieve optimal patient outcomes.

## Acknowledgments

We would like to thank the patient and their family for participating in this study.

## Author contributions

**Conceptualization:** Bayyena Abu-Radwan, Haya Abu Mayyaleh, Islam Jadallah, Mohammed Saleh.

**Data curation:** Bayyena Abu-Radwan, Haya Abu Mayyaleh, Islam Jadallah, Mohammed Saleh.

**Investigation:** Islam Jadallah, Mohammed Saleh.

**Resources:** Ahmad Fasfoos.

**Software:** Bayyena Abu-Radwan, Haya Abu Mayyaleh.

**Supervision:** Yousef Abu Asbeh.

**Validation:** Ahmad Fasfoos.

**Visualization:** Islam Jadallah, Mohammed Saleh.

**Writing – original draft:** Bayyena Abu-Radwan, Haya Abu Mayyaleh, Islam Jadallah, Mohammed Saleh.

**Writing – review & editing:** Bayyena Abu-Radwan, Haya Abu Mayyaleh, Islam Jadallah, Mohammed Saleh.

## References

[1] Gagnier JJ Kienle G Altman DG Moher D Sox H Riley D. The CARE guidelines: consensus-based clinical case reporting guideline development. Global Adv Health Med. 2013;2:38–43. 10.7453/gahmj.2013.008 PMC3833570 24416692

[2] Gessese AT. Review on epidemiology and public health significance of hydatidosis. Vet Med Int. 2020;2020:8859116. 33354312 10.1155/2020/8859116 PMC7735834

[3] Al-Jawabreh A Ereqat S Dumaidi K. The clinical burden of human cystic echinococcosis in Palestine, 2010-2015. PLoS NeglTrop Dis. 2017;11:e0005717. 10.1371/journal.pntd.0005717 PMC5510903 28672021

[4] Wen H Vuitton L Tuxun T. Echinococcosis: advances in the 21st Century. Clin Microbiol Rev. 2019;32:e00075–18. 30760475 10.1128/CMR.00075-18 PMC6431127

[5] Hamamci EO Besim H Korkmaz A. Unusual locations of hydatid disease and surgical approach. ANZ J Surg. 2004;74:356–60. 15144257 10.1111/j.1445-1433.2004.02981.x

[6] Tartar T Bakal U Sarac M Kazez A. Laboratory results and clinical findings of children with hydatid cyst disease. Niger J Clin Pract. 2020;23:1008–12. 32620733 10.4103/njcp.njcp_531_19

[7] Durakbasa CU Tireli GA Sehiralti V Sander S Tosyali AN Mutus M. An audit on pediatric hydatid disease of uncommon localization: incidence, diagnosis, surgical approach, and outcome. J Pediatr Surg. 2006;41:1457–63. 16863854 10.1016/j.jpedsurg.2006.04.024

[8] Lawandi A Yansouni CP Libman M. A 9-year-old female with a cough and cavitary lung lesion. Clin Infect Dis. 2019;69:705–8. 31986208 10.1093/cid/ciy769

[9] Masterton RG O’Doherty MJ Eykyn SJ. *Streptococcus milleri* infection of a hepatopulmonary hydatid cyst. Eur J Clin Microbiol. 1987;6:414–5. 3665896 10.1007/BF02013097

[10] Khasawneh RA Mohaidat ZM Khasawneh RA Zoghoul SB Henawi YM. Unusual intramuscular locations as a first presentation of hydatid cyst disease in children: a report of two cases. BMC Pediatr. 2021;21:371. 34465295 10.1186/s12887-021-02843-5 PMC8406844

[11] Rawat S Kumar R Raja J Singh RS Thingnam SKS. Pulmonary hydatid cyst: review of literature. J Family Med Primary Care. 2019;8:2774–8. 31681642 10.4103/jfmpc.jfmpc_624_19 PMC6820383

[12] Kayhan S Akgüneş A. Histopathologically diagnosed pulmonary complicated hydatid cyst cases. Turkiye Parazitol Derg. 2011;35:189–93. 22198916 10.5152/tpd.2011.49

[13] Tartar T Bakal U Sarac M Akdeniz I Kazez A. Primary urachal hydatid cyst in a child: a case report. Iran J Parasitol. 2019;14:352–5. 31543926 PMC6737373

[14] Alshoabi SA Alkalady AH Almas KM. Hydatid disease: a radiological pictorial review of a great neoplasms Mimicker. Diagnostics (Basel, Switzerland). 2023;13:1127. 36980435 10.3390/diagnostics13061127 PMC10047450

[15] Polat P Kantarci M Alper F Suma S Koruyucu MB Okur A. Hydatid disease from head to toe. Radiographics. 2003;23:475–94; quiz 536. 12640161 10.1148/rg.232025704

[16] Sayek I Tirnaksiz MB Dogan R. Cystic hydatid disease: current trends in diagnosis and management. Surg Today. 2004;34:987–96. 15580379 10.1007/s00595-004-2830-5

[17] Usluer O Ceylan KC Kaya S Sevinc S Gursoy S. Surgical management of pulmonary hydatid cysts: is size an important prognostic indicator? Tex Heart Inst J. 2010;37:429–34. 20844615 PMC2929855

[18] AlRashed FM AlShahrani DA. Infected pulmonary hydatid cyst: a challenging diagnosis. Saudi Med J. 2024;45:433–6. 38657978 10.15537/smj.2024.45.4.20230078 PMC11147576

[19] Işik S Sözmen SC Güzeloğlu E Öztürk T Asilsoy S. Pulmonary hydatid cyst disease mimicking necrotizing pneumonia in a child with leukocytoclastic vasculitis. Turk Pediatr Ars. 2018;53:117–9. 10.5152/TurkPediatriArs.2018.3670 PMC6089786 30116133

